# A rapid minor groove binder PCR method for distinguishing the vaccine strain *Brucella abortus* 104M

**DOI:** 10.1186/s12917-018-1350-2

**Published:** 2018-01-24

**Authors:** Wenlong Nan, Lide Qin, Yong Wang, Yueyong Zhang, Pengfei Tan, Yuqi Chen, Kairong Mao, Yiping Chen

**Affiliations:** 1 Laboratory of Diagnositics Development, China Animal Health and Epidemiology Center, 369 Nanjing Road, Qingdao, Shandong 266032 China; 2 College of Veterinary Medicine, Yangzhou University, 12 East Wenhui Road, Yangzhou, Jiangsu 225009 China; 3 China Institute of Veterinary Drug Control, 8 Zhongguanchun South Street, Beijing, 100081 China; 4 Xi’an Jiaotong-Liverpool University, 111 Ren’ai Road, Suzhou, Jiangsu Province, 215123 China

**Keywords:** Brucellosis, *Brucella abortus*, Minor groove binder, SNP-based assay

## Abstract

**Background:**

Brucellosis is a widespread zoonotic disease caused by Gram-negative *Brucella* bacteria. Immunisation with attenuated vaccine is an effective method of prevention, but it can interfere with diagnosis. Live, attenuated *Brucella abortus* strain 104M has been used for the prevention of human brucellosis in China since 1965. However, at present, no fast and reliable method exists that can distinguish this strain from field strains. Single nucleotide polymorphism (SNP)-based assays offer a new approach for such discrimination. SNP-based minor groove binder (MGB) and Cycleave assays have been used for rapid identification of four *Brucella* vaccine strains (*B. abortus* strains S19, A19 and RB51, and *B. melitensis* Rev1). The main objective of this study was to develop a PCR assay for rapid and specific detection of strain 104M.

**Results:**

We developed a SNP-based MGB PCR assay that could successfully distinguish strain 104M from 18 representative strains of *Brucella* (*B. abortus* biovars 1, 2, 3, 4, 5, 6, 7 and 9, *B. melitensis* biovars 1, 2 and 3, *B. suis* biovars 1, 2, 3 and 4, *B. canis*, *B. neotomae*, and *B. ovis*), four *Brucella* vaccine strains (A19, S19, S2, M5), and 55 *Brucella* clinical field strains. The assay gave a negative reaction with four non-*Brucella* species (*Escherichia coli*, *Pasteurella multocida*, *Streptococcus suis* and *Pseudomonas aeruginosa*). The minimum sensitivity of the assay, evaluated using 10-fold dilutions of chromosomal DNA, was 220 fg for the 104M strain and 76 fg for the single non-104M *Brucella* strain tested (*B. abortus* A19). The assay was also reproducible (intra- and inter-assay coefficients of variation = 0.006–0.022 and 0.012–0.044, respectively).

**Conclusions:**

A SNP-based MGB PCR assay was developed that could straightforwardly and unambiguously distinguish *B. abortus* vaccine strain 104M from non-104M *Brucella* strains. Compared to the classical isolation and identification approaches of bacteriology, this real-time PCR assay has substantial advantages in terms of simplicity and speed, and also reduces potential exposure to live *Brucella*. The assay developed is therefore a simple, rapid, sensitive, and specific tool for brucellosis diagnosis and control.

## Background

Brucellosis is a widespread zoonotic disease caused by various Gram-negative *Brucella* bacterial species that damages human health and results in considerable economic losses. Annually, more than 500,000 new human brucellosis cases are reported worldwide [1], and cases have increased rapidly over the last decade in all provinces in China [2]. Human brucellosis is transmitted by eating contaminated food products of animal origin and via direct animal contact [2], and although rarely fatal, it can be severely debilitating and disabling [3].

For over a century, vaccination and the culling of animals has been performed to control this disease [4–6]. In China, live attenuated *Brucella* strains are widely used for the prevention and control of brucellosis, including *Brucella abortus* strain A19 in cattle, *B. suis* S2 used in swine, and *B. melitensis* M5 in sheep and goat. In addition, live, attenuated *B. abortus* 104M has been adopted as a vaccine for use in humans since 1965. This strain, which was first isolated from the foetus of an aborted cow in the former Soviet republic in 1950, exhibits low virulence, high stability and high immuno-antigenicity [7].

However, since it is a live attenuated strain, vaccination with 104M may cause vaccine-related cases of brucellosis, and it may be difficult to differentiate between a vaccine response and a natural infection, which complicates diagnosis. At present, a rapid and reliable method for distinguishing 104M from field strains is not available. The main objective of this study was to develop a PCR assay for rapid and specific detection of 104M.

## Methods

### Strains and DNA extraction

*Brucella* strains used in the present study are listed in Table 1. These comprised 18 representative strains of *Brucella* species and biovars, five *Brucella* vaccine strains, and 55 *Brucella* field strains. In addition, four non-target organisms (*Escherichia coli* K99, *Pasteurella multocida* C48–1, *Pseudomonas aeruginosa* DI-1, and *Streptococcus suis* ST171) were included. *Brucella* strains were cultured on tryptose agar at 37 °C with 5–10% CO_2_ when required for 48–72 h in a biosafety level 3-equipped laboratory. Bacteria were then washed with normal saline containing 0.5% formaldehyde, and inactivated at 37 °C for 24 h. The four non-*Brucella* species were cultivated as described previously [8], and harvested and inactivated as described above. Unless specified, genomic DNA was extracted with the QIAamp DNA mini kit according to the manufacturer’s instructions (Qiagen GmbH., D40724 Hilden).

**Table 1:** *Brucella spp*. strains used in the present study

Species (biovar)	Strain	Type	Host	Region
*B. abortus* (1)	A544 (CVCC790, ATCC23448)	Reference strain	Bovine	United Kingdom
*B. abortus* (2)	86/8/59 (CVCC12, ATCC23449)	Reference strain	Bovine	United Kingdom
*B. abortus* (3)	Tulya (CVCC13, ATCC23450)	Reference strain	Bovine	United Kingdom
*B. abortus* (4)	292 (CVCC16, ATCC23451)	Reference strain	Bovine	United Kingdom
*B. abortus* (5)	B3196 (CVCC14, ATCC23452)	Reference strain	Bovine	United Kingdom
*B. abortus* (6)	870 (CVCC17, ATCC23453)	Reference strain	Bovine	United Kingdom
*B. abortus* (7)	63/75 (CVCC15, ATCC23454)	Reference strain	Bovine	United Kingdom
*B. abortus* (9)	C68 (CVCC11, ATCC23455)	Reference strain	Bovine	United Kingdom
*B. abortus* (4)	C72–62 (CVCC887)	Field strain	Bovine	Inner Mongolia
*B. abortus* (4)	C72–63 (CVCC888)	Field strain	Bovine	Inner Mongolia
*B. abortus* (4)	C72–61 (CVCC886)	Field strain	Bovine	Inner Mongolia
*B. abortus* (Unknown)	SHDeer-74 (CVCC780)	Field strain	Cervine	Shanghai
*B. abortus* (Unknown)	C72–387 (CVCC785)	Field strain	Bovine	Heilongjiang
*B. abortus* (Unknown)	C72–10 (CVCC786)	Field strain	Bovine	Heilongjiang
*B. abortus* (Unknown)	2308 (CVCC788)	Field strain	Bovine	Heilongjiang
*B. abortus* (Unknown)	HBCow-1 (CVCC2408)	Field strain	Bovine	Hubei
*B. abortus* (Unknown)	HBCow-2 (CVCC2409)	Field strain	Bovine	Hubei
*B. abortus* (Unknown)	C72–12 (CVCC3621)	Field strain	Bovine	Heilongjiang
*B. abortus* (Unknown)	C72–8401 (CVCC3622)	Field strain	Bovine	Inner Mongolia
*B. abortus* (Unknown)	C72–8403 (CVCC3623)	Field strain	Bovine	Inner Mongolia
*B. abortus* (Unknown)	NMCow-2 (CVCC3635)	Field strain	Bovine	Inner Mongolia
*B. melitensis* (1)	16 M (CVCC70002, ATCC23456)	Reference strain	Caprine	United Kingdom
*B. melitensis* (2)	63/9 (CVCC21, ATCC23457)	Reference strain	Caprine	United Kingdom
*B. melitensis* (3)	Ether (CVCC20, ATCC23458)	Reference strain	Caprine	United Kingdom
*B. melitensis* (1)	Goat-901 (CVCC3627)	Field strain	Caprine	Inner Mongolia
*B. melitensis* (Unknown)	CVCC3620	Field strain	Unknown	Inner Mongolia
*B. melitensis* (Unknown)	C71–1257 (CVCC928)	Field strain	Caprine	Inner Mongolia
*B. melitensis* (Unknown)	C71–13 (CVCC929)	Field strain	Caprine	Inner Mongolia
*B. melitensis* (Unknown)	C71–35 (CVCC936)	Field strain	Ovine	Qinghai
*B. melitensis* (Unknown)	C71–44 (CVCC938)	Field strain	Caprine	Xinjiang
*B. melitensis* (Unknown)	Goat-963 (CVCC952)	Field strain	Caprine	Inner Mongolia
*B. melitensis* (Unknown)	M54–8 (CVCC3624)	Field strain	Ovine	Qinghai
*B. melitensis* (Unknown)	Goat-866 (CVCC3625)	Field strain	Caprine	Inner Mongolia
*B. melitensis* (Unknown)	Goat-872 (CVCC3626)	Field strain	Caprine	Inner Mongolia
*B. melitensis* (Unknown)	Goat-865 (CVCC3628)	Field strain	Caprine	Inner Mongolia
*B. suis* (1)	S1330 (CVCC70524, ATCC23444)	Reference strain	Porcine	United Kingdom
*B. suis* (2)	Thomsen (CVCC22, ATCC23445)	Reference strain	Porcine	United Kingdom
*B. suis* (3)	686 (CVCC23, ATCC23446)	Reference strain	Porcine	United Kingdom
*B. suis* (4)	40 (CVCC24, ATCC23447)	Reference strain	Porcine	United Kingdom
*B. suis* (3)	KP6 (CVCC3651)	Field strain	Porcine	Guangdong
*B. suis* (3)	ZC5 (CVCC3653)	Field strain	Porcine	Guangdong
*B. suis* (3)	ZC1 (CVCC3655)	Field strain	Porcine	Guangdong
*B. suis* (3)	ZC6 (CVCC3649)	Field strain	Porcine	Guangdong
*B. suis* (3)	KP1 (CVCC3658)	Field strain	Porcine	Guangdong
*B. suis* (3)	KP2 (CVCC3659)	Field strain	Porcine	Guangdong
*B. suis* (3)	KP3 (CVCC3660)	Field strain	Porcine	Guangdong
*B. suis* (3)	KP5 (CVCC3661)	Field strain	Porcine	Guangdong
*B. suis* (3)	HNPig-1 (CVCC3662)	Field strain	Porcine	Hainan
*B. suis* (3)	HNPig-2 (CVCC3663)	Field strain	Porcine	Hainan
*B. suis* (Unknown)	BS4 (CVCC1072)	Field strain	Porcine	Russian
*B. suis* (Unknown)	C73–5 (CVCC1080)	Field strain	Porcine	Guangxi
*B. suis* (Unknown)	C73–10 (CVCC1083)	Field strain	Porcine	Guangxi
*B. suis* (Unknown)	C73–11 (CVCC1084)	Field strain	Porcine	Guangxi
*B. suis* (Unknown)	C73–13 (CVCC1085)	Field strain	Porcine	Guangxi
*B. suis* (Unknown)	C73–23 (CVCC1089)	Field strain	Porcine	Guangxi
*B. suis* (Unknown)	C73–25 (CVCC1091)	Field strain	Porcine	Guangxi
*B. suis* (Unknown)	C73–26 (CVCC1092)	Field strain	Porcine	Guangxi
*B. suis* (Unknown)	Br.63/3 (CVCC3639)	Field strain	Unknown	United Kingdom
*B. suis* (Unknown)	Br.63/142 (CVCC3640)	Field strain	Unknown	United Kingdom
*B. suis* (Unknown)	Br.86/27 (CVCC3641)	Field strain	Unknown	United Kingdom
*B. suis* (Unknown)	Br.63/62 (CVCC3642)	Field strain	Unknown	United Kingdom
*B. suis* (Unknown)	Br.79/224 (CVCC3643)	Field strain	Unknown	United Kingdom
*B. suis* (Unknown)	Br.Thomsen1720 (CVCC3644)	Field strain	Unknown	United Kingdom
*B. suis* (Unknown)	Br.Thomsen5 (CVCC3645)	Field strain	Unknown	United Kingdom
*B. suis* (Unknown)	Br.63/225 (CVCC3646)	Field strain	Unknown	United Kingdom
*B. suis* (Unknown)	Br.63/32 (CVCC3647)	Field strain	Unknown	United Kingdom
*B. suis* (Unknown)	Br.64/24 (CVCC3648)	Field strain	Unknown	United Kingdom
*B. suis* (Unknown)	ZC2 (CVCC3656)	Field strain	Porcine	Guangdong
*B. suis* (Unknown)	ZC3 (CVCC3657)	Field strain	Porcine	Guangdong
*B. suis* (Unknown)	DF1 (CVCC3654)	Field strain	Porcine	Guangdong
*B. suis* (Unknown)	SD1 (CVCC3652)	Field strain	Porcine	Guangdong
*B. suis* (Unknown)	ZC4 (CVCC3650)	Field strain	Porcine	Guangdong
*B. ovis*	63/290 (CVCC70015, ATCC25840)	Reference strain	Ovine	United Kingdom
*B. canis*	RM6/66 (CVCC70701, ATCC23365)	Reference strain	Canine	United Kingdom
*B. canis*	KP4 (CVCC3664)	Field strain	Canine	Guangdong
*B. neotomae*	5 K33 (CVCC70721, ATCC23459)	Reference strain	Unknown	United Kingdom
*B. abortus* (1)	A19	Vaccine	–	–
*B. melitensis* (1)	M5	Vaccine	–	–
*B. abortus* (Unknown)	104 M	Vaccine	–	–
*B. abortus* (1)	S19	Vaccine	–	–
*B. suis* (1)	S2	Vaccine	–	–

Strains were identified and provided by the China Veterinary Culture Collection Centre (CVCC)

Unknown = unknown biovar or host

### Minor groove binder (MGB) PCR

In this real-time PCR assay, a pair of short TaqMan 5′- labelled, 3′- MGB probes defining the single nucleotide polymorphism (SNP) were used to interrogate the 104M strain and non-104 M *Brucella* strains. Use of the MGB protein raises the melting temperature of probes meaning that a single base mismatch causes more destabilisation than would be the case with a longer probe [9]. This facilitates accurate SNP detection. For distinguishing *B. abortus* 104M, the SNP C_228_–T_228_ in NL70_10085 was selected. This SNP was identified by comparison of the *B. abortus* 104M draft genomic sequence with the sequences of *B. abortus* 9–941, *B. melitensis* M28, *B. suis* S1330, *B. canis* ATCC 23365, and *B. ovis* ATCC 25840, *B. pinnipedialis* B2/94, *B. microti* CCM 4915. One set of primers and probes was designed based on this SNP (Table 2).

**Table 2:** Targets, primers and probes used for the MGB PCR assay with the associated working concentrations

Target (positon)	Gene description	Working concentration (nM)	
		Probe	Primer
NL70_10085 (228)	molecular chaperone DnaK	VAC: CCGTCG**T**TATGACGAT (160)	F: CCGGAAGGCACCCTTTTT (600)
		NON: CCGTCG**C**TATGACGA (400)	R: GATCCTTGTCCTTGGTGACCAT (600)

The position of the SNP within each target is shown in parentheses. The target SNP is shown in bold underlined font in both vaccine (VAC) and nonspecific (NON) probes

The assay was performed using the TransStart Green qPCR SuperMix kit (TransGen Biotech Co., Beijing, China) in a reaction volume of 25 μL containing a reaction mixture volume of 12.5 μL with the working concentrations of primers and probes listed in Table 2, together with the DNA template (2 μL). For detection of the 104M strain, the VAC probe was labelled with 6-carboxyfluorescein (FAM) at the 5′-end and MGB eclipse at the 3′-end. For detection of non-104 M *Brucella* strains, the NON probe was labelled with the fluorophore 4,7,2′-trichloro-7′-phenyl-6-carboxyfluorescein (VIC) at the 5′-end and MGB eclipse at the 3′-end.

PCR cycling parameters were as follows: 95 °C for 3 min, followed by 40 cycles of 95 °C for 5 s, 56 °C for 10 s, and 72 °C for 10 s. Amplification was performed using the Bio-Rad MiniOpticon system (Bio-Rad Laboratories, Inc., Hercules, CA).

### Sensitivity, specificity, and reproducibility

For assay sensitivity tests, the minimum detection limit of MGB PCR was evaluated using 10-fold serial dilutions of genomic DNA from *B. abortus* 104M for FAM fluorescence, and *B. abortu*s A19 for VIC fluorescence. Each dilution was included in the assay to determine the minimum discriminatory amount of genomic DNA detected in the assay.

For assay specificity tests, we evaluated whether MGB PCR could distinguish the 104M strain from common species and other vaccine strains of *Brucella* using the representative and vaccine *Brucella* strains listed in Table 1. These strains included almost all common species and biovars of *Brucella* and the vaccine strains currently used in China. The four non-*Brucella* spp. (*Escherichia coli* K99, *Pasteurella multocida* C48–1, *Streptococcus suis* ST171, and *Pseudomonas aeruginosa* DI-1) were also tested.

Assay reproducibility was determined by calculating the intra- and inter-assay coefficients of variation (CV), using at least three replicates of each of the 10-fold serial dilutions of genomic DNA to generate a standard curve. The efficiency of the assay was determined using the following calculation: Efficiency = 10 (− 1/slope) – 1.

### Detection of clinical field strains

A further 55 *Brucella spp*. field isolates (see Table 1) that were isolated from different animal species and areas, identified and provided by China Veterinary Culture Collection Centre were also tested.

## Results

### Assay sensitivity

Using 10-fold serial dilutions of *B. abortus* 104M genomic DNA ranging from 1.1 ng/μL to 0.11 fg/μL, the minimum discriminatory sensitivity for detection of 104M–specific strains was ~ 220 fg per reaction for MGB PCR (Table 3). Similarly, using 10-fold serial dilutions of *B. abortus* A19 genomic DNA ranging from 3.8 ng/μL to 0.38 fg/μL, the minimum discriminatory sensitivity for detection of non-104 M *Brucella* strains was ~ 76 fg per reaction for MGB PCR (Table 3). These results indicated that the assays were highly sensitive for the detection of 104M and non-104 M *Brucella* genomic DNA in a single reaction.

**Table 3:** Mean quantification cycle (Cq) values resulting from MGB PCR^a^

Detection of 104M genomic DNA			Detection of A19 genomic DNA		
Concentration	Cq of FAM	Cq of VIC	Concentration	Cq of FAM	Cq of VIC
1.1 ng/μL	24.06	NA	3.8 ng/μL	NA	17.49
110 pg/μL	27.20	NA	380 pg/μL	NA	20.89
11 pg/μL	30.92	NA	38 pg/μL	NA	24.39
1.1 pg/μL	35.27	NA	3.8 pg/μL	NA	31.90
110 fg/μL	38.25	NA	380 fg/μL	NA	34.69
11 fg/μL	NA	NA	38 fg/μL	NA	38.11
1.1 fg/μL	NA	NA	3.8 fg/μL	NA	NA
0.11 fg/μL	NA	NA	0.38 fg/μL	NA	NA

^a^FAM = 6-carboxyfluorescein; VIC = 4,7,2′-trichloro-7′-phenyl-6-carboxyfluorescein; NA = not applicable

### Assay specificity

**Fig. 1:**
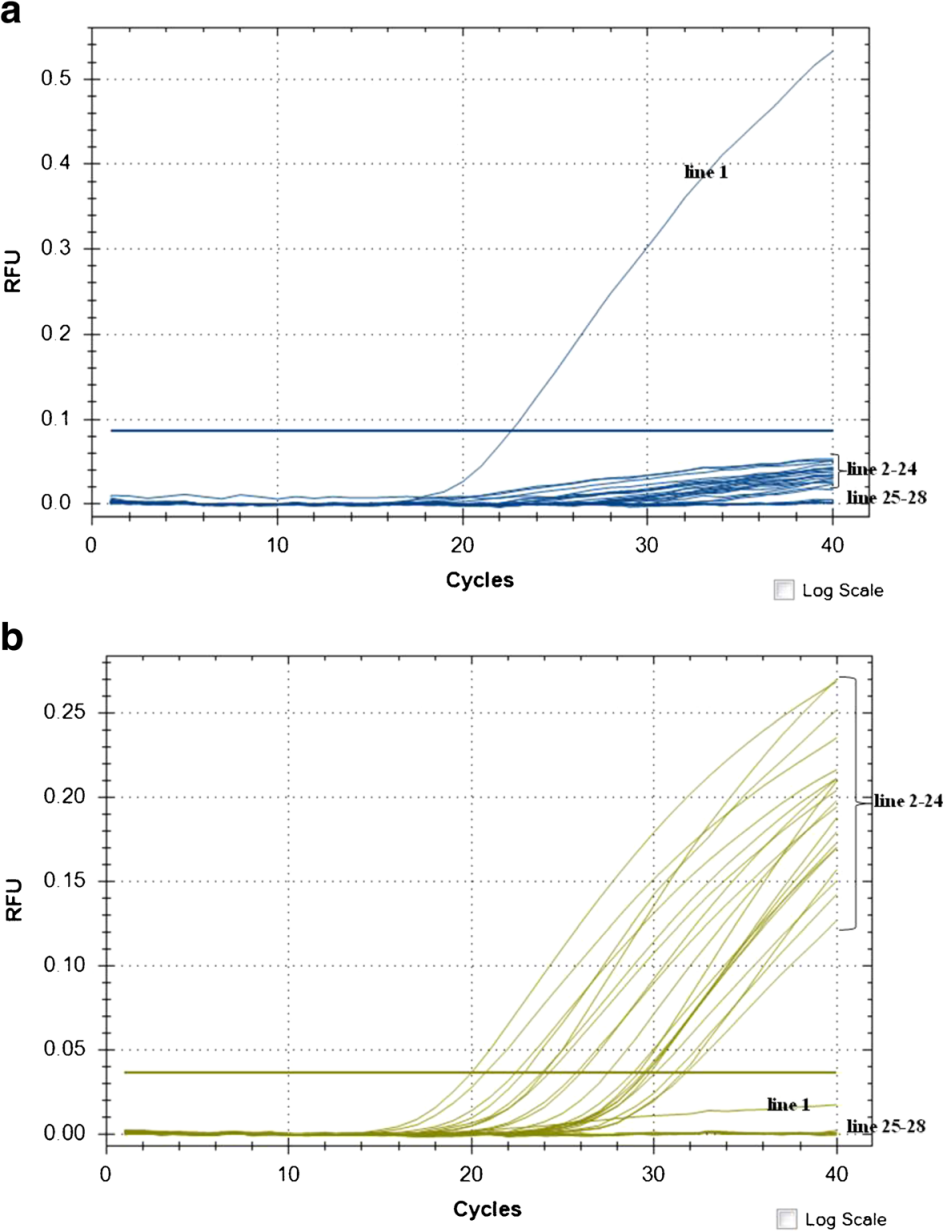
Specificity of the MGB PCR assay using representative experiments for the detection of (**a**) FAM fluorescence and (**b**) VIC fluorescence. The following strains were subjected to amplification assays: Line 1, 104 M; Line 2 *Brucella suis* biovar 1 S1330; Line 3, *B. suis* biovar 2 Thomsen; Line 4, *B. suis* biovar 3686; Line 5, *B. suis* biovar 4 40; Line 6, *B. abortus* biovar 1 A544; Line 7, *B. abortus* biovar 2 86/8/59; Line 8, *B. abortus* biovar 3 Tulya; Line 9, *B. abortus* biovar 4292; Line 10, *B. abortus* biovar 5 B3196; Line 11, *B. abortus* biovar 6870; Line 12, *B. abortus* biovar 7 63/75; Line 13, *B. abortus* biovar 9 C68; Line 14, *B. melitensis* biovar 1 16 M; Line 15, *B. melitensis* biovar 2 63/9; Line 16, *B. melitensis* biovar 3442; Line 17, *B. ovis* 63/290; Line 18, *B. canis* RM6/66; Line 19, *B. neotomae* 5 K33; Line 20, S2; Line 21, S19; Line 22, A19; Line 23, M5; Line 24, S2; Line 25, *Escherichia coli* K99; Line 26, *Pasteurella multocida* C48–1; Line 27, *Streptococcus suis* ST171; Line 28, *Pseudomonas aeruginosa* DI-1

For evaluating specificity, the representative and vaccine *Brucella* strains listed in Table 1 were tested using the MGB PCR method described. The results showed that 18 representative strains of *Brucella* (*B. abortus* biovars 1, 2, 3, 4, 5, 6, 7 and 9, *B. melitensis* biovars 1, 2 and 3, *B. suis* biovars 1, 2, 3 and 4, *B. canis*, *B. neotomae* and *B. ovis*), and four *Brucella* vaccine strains (A19, S19, S2, M5) gave strong VIC fluorescence and weak FAM fluorescence below the threshold detection level (Fig. 1a and b).

Only the *B. abortus* 104M vaccine strain gave strong FAM fluorescence and weak VIC fluorescence below the threshold detection level (Fig. 1a and b), indicating that the assay was 104M–specific. All four non-*Brucella* species (*Escherichia coli* K99, *Pasteurella multocida* C48–1, *Streptococcus suis* ST171 and *Pseudomonas aeruginosa* DI-1) were negative for both FAM and VIC fluorescence. These results suggest the MGB PCR assay was highly capable of differentiating 104M from non-104 M *Brucella* isolates and non-*Brucella* strains.

### Assay reproducibility

The standard curve generated using genomic DNA was linear over a wide range of dilutions (R^2^ = 0.997 and slope = − 3.645 for FAM fluorescence; R2 = 0.982 and slope = − 4.342 for VIC fluorescence). The assay was reproducible, with intra-assay CVs ranging from 0.006 to 0.022, and inter-assay CVs of 0.012 to 0.044. The efficiency of the assay was 88.1% for FAM fluorescence and 69.9% for VIC fluorescence. These figures were used to determine the threshold for detection.

### Detection of clinical field strains

The results demonstrated strong VIC fluorescence and weak FAM fluorescence for all 55 *Brucella spp*. field isolates tested (Table 1), indicating that none were the 104M strain.

## Discussion

The *B. abortus* vaccine strain 104M is a stable antigenic structure with low virulence and high immunogenicity, hence it has been used in China since 1965 to vaccinate cattle and humans against brucellosis. Because using a live vaccine may lead to severe pathogenic injury associated with allergy, the 104M strain was only recommended for high-risk populations in China [7], such as those at high risk due to their occupation [10]. The scratch vaccination method was used to introduce five billion bacteria, which achieved 90% protection for a 12 month duration [7]. In some areas in China, vaccination intervention in humans had an obvious effect; the reported cases of brucellosis in the Arong Banner declined sharply by 84.17% from 2005 to 2006 following vaccination, and the morbidity rate of brucellosis declined from 34,732 per 100,000 to 5454 per 100,000 [11].

However, due to lack of serological differentiation, it is difficult to distinguish 104M by serological assay alone. The recent development of SNP-based real-time assays offers a new approach for overcoming this hurdle. SNP-based MGB and Cycleave assays have been used for rapid identification of four *Brucella* vaccine strains (*B. abortus* strains S19, A19 and RB51, and *B. melitensis* Rev1) [12, 13].

In the present study, we developed a new MBG PCR assay that can successfully distinguish 104M strains from other bacterial strains, with a sensitivity of 220 fg, equating to around 60 cells. Furthermore, our MGB PCR assay can detect non-104 M *Brucella* strains in a single reaction with a sensitivity of 76 fg, equating to around 30 cells. This assay allows accurate and reliable discrimination of 104M and non-104 M *Brucella* strains from common species and biovars of *Brucella*, *Brucella* vaccines, and other bacterial strains. Our assay therefore provides a simple, rapid, sensitive, and specific tool for use in the control of brucellosis.

## Conclusions

A SNP-based MGB PCR assay was developed that could straightforwardly and unambiguously distinguish *B. abortus* vaccine strain 104M. Results of our study indicate the assay allows accurate and reliable discrimination of 104M and non-104 M *Brucella* strains from common species and biovars of *Brucella*, *Brucella* vaccines, and other bacterial strains. The minimum detection limit of the assay was 220 fg for strain 104M and 76 fg for the single non-104 M *Brucella* strain tested. Compared to the classical isolation and identification approaches of bacteriology, this real-time PCR assay has substantial advantages in terms of simplicity and speed, and also reduces the potential for exposure to live *Brucella*. As real-time PCR instruments become more widely used in China, the approach will become widely applicable in routine diagnostics. The assay developed is therefore a simple, rapid, sensitive, and specific tool for brucellosis diagnosis and control.

## Abbreviations

*B. abortus*: *Brucella abortus*
*B. canis*: *Brucella canis*
*B. melitensis*: *Brucella melitensis*
*B. neotomae*: *Brucella neotomae*
*B. ovis*: *Brucella ovis*
*B. suis*: *Brucella suis*
FAM: 6-carboxyfluorescein
MGB: Minor groove binder
SNP: Single nucleotide polymorphism-based
VIC: 4,7,2′-trichloro-7′-phenyl-6-carboxyfluorescein
